# What Can We Learn from a Metagenomic Analysis of a Georgian Bacteriophage Cocktail?

**DOI:** 10.3390/v7122958

**Published:** 2015-12-12

**Authors:** Henrike Zschach, Katrine G. Joensen, Barbara Lindhard, Ole Lund, Marina Goderdzishvili, Irina Chkonia, Guliko Jgenti, Nino Kvatadze, Zemphira Alavidze, Elizabeth M. Kutter, Henrik Hasman, Mette V. Larsen

**Affiliations:** 1 Center for Biological Sequence Analysis, Department of Systems Biology, Technical University of Denmark, 2800 Kgs. Lyngby, Denmark; henrike@cbs.dtu.dk (H.Z.); b.lindhard@live.dk (B.L.); lund@cbs.dtu.dk (O.L.); henh@ssi.dk (H.H.); 2 National Food Institute, Technical University of Denmark, 2800 Kgs. Lyngby, Denmark; kagjo@food.dtu.dk; 3 Eliava Institute of Bacteriophages, Microbiology and Virology, 3 Gotua Str., Tbilisi 0160, Georgia; mgoderdzishvili@gtu.ge (M.G.); irinachkonia@yahoo.com (I.C.); gulikojgenti@yahoo.com (G.J.); ninqvatadze@yahoo.com (N.K.); 4 Eliava Biopreparations LTD, 3 Gotua Str., Tbilisi 0160, Georgia; z.i.alavidze@gmail.com; 5 Lab 1, The Evergreen State College, Olympia, WA 98505, USA; KutterB@evergreen.edu

**Keywords:** phage therapy, Eliava Intestiphage, whole genome sequence analysis, metagenomics

## Abstract

Phage therapy, a practice widespread in Eastern Europe, has untapped potential in the combat against antibiotic-resistant bacterial infections. However, technology transfer to Western medicine is proving challenging. Bioinformatics analysis could help to facilitate this endeavor. In the present study, the Intesti phage cocktail, a key commercial product of the Eliava Institute, Georgia, has been tested on a selection of bacterial strains, sequenced as a metagenomic sample, *de novo* assembled and analyzed by bioinformatics methods. Furthermore, eight bacterial host strains were infected with the cocktail and the resulting lysates sequenced and compared to the unamplified cocktail. The analysis identified 23 major phage clusters in different abundances in the cocktail, among those clusters related to the ICTV genera *T4likevirus*, *T5likevirus*, *T7likevirus*, *Chilikevirus* and *Twortlikevirus*, as well as a cluster that was quite distant to the database sequences and a novel *Proteus* phage cluster. Examination of the depth of coverage showed the clusters to have different abundances within the cocktail. The cocktail was found to be composed primarily of *Myoviridae* (*35*%) and *Siphoviridae* (32%), with *Podoviridae* being a minority (15%). No undesirable genes were found.

## 1. Introduction

Antibiotic resistance in human pathogenic bacteria is a threat to public health that has grown immensely in the last years. The World Health Organization (WHO) recognized the severity of the problem in two reports made public in 2012 and 2014, stating that “A post-antibiotic era—in which common infections and minor injuries can kill—far from being an apocalyptic fantasy, is instead a very real possibility for the 21st Century” [1]. It is therefore all the more urgent to secure alternative treatment strategies. Phage therapy is one of the alternatives to antibiotics that for a long time has been underexplored in Western medicine. Bacteriophages, viruses of bacteria, have been employed to combat bacterial infections in certain Eastern European countries since the mid-1920s [2,3]. With the number of phages on earth estimated at 10^31^ in total [4], they are the most abundant entity in the biosphere and, as natural predators of bacteria, they hold largely untapped therapeutic potential [5].

During the Soviet era, antibiotics were not readily available in the USSR, which contributed to the widespread use of phages for treatment of various sorts of bacterial infections [6]. In particular, the George Eliava Institute in Tbilisi, Georgia, founded in 1923, has more than 90 years of experience in employing phages for treatment of bacterial infections in humans, either as single preparations or in mixtures, *i.e.*, phage cocktails.

Phage therapy is largely regarded as safe and effective in those countries where it is still practiced [7,8,9,10]. This is reinforced by the long-standing tradition of its use. The enormous body of experience with clinical phage therapy, which has primarily been reported in non-English languages [11], is now more and more being made available to the scientific community thanks to the concerted efforts of Elizabeth Kutter, Jan Borysowski, Harald Brüssow, Ryszard Międzybrodzki, Andrzej Górski, Beata Weber-Dąbrowska, Mzia Kutateladze, Zemphira Alavidze, Marina Goderdzishvili, Revaz Adamia and others [8,9].

Additionally, a number of more recent trials have been carried out in accordance to the strict guidelines demanded by legislative bodies and published, notably two T4 oral application safety trials [12,13], a trial of *Pseudomonas aeruginosa* phages for treatment of chronic otitis [14], a phase I trial of phage therapy for venous leg ulcers [15] and a trial of Russian phage cocktail administration in healthy individuals [16].

Despite the growing body of evidence on the safety and efficacy of phage therapy, the technology proves hard to transfer despite considerable interest by Western researchers. One of the challenges is a lack of definition and characterization of the phages used, as the exact composition of phages in the cocktails produced in Eastern Europe is largely unknown [17]. Advances in metagenomics and decreasing sequencing costs have made it possible to analyze mixed phage samples without the need to separate the component phages. This is especially essential when the specific bacterial hostsstrains are unknown and the phages can thus not be individually propagated for traditional analysis. This metagenomic approach was first used for marine viral communities in 2002 [18]. One of the latest milestones in this endeavor consists of a metagenomic study of a Russian phage cocktail as well as a safety trial, performed by McCallin *et al.* in 2013 [16].

Here, we present a metagenomic analysis of the longest-used such commercial phage cocktail in the world, still routinely employed for human therapy in the Republic of Georgia. Intesti bacteriophage was created at the Pasteur Institute, Paris by Felix d’Herelle [19] as a multi-component treatment and prophylaxis of intestinal infections. From early on, the preparation is a combination of phage active against *Shigella*, *Escherichia*, *Salmonella*, *Enterococcus*, *Staphylococcus*, *Streptococcus* and *Pseudomonas*. Its advantages lie in its activity against a wide variety of enteric bacteria, allowing it to be used empirically during the first days of gastrointestinal illness, before the microbiological culture results are in, along with its frequent ability to help restore balance to the gut microbiome even where no explicit pathogen has been identified as the cause of the problem.

Intesti bacteriophage was first used clinically in Georgia in 1937 by S. Mikeladze [20]. Already in 1938, M.N. Luria used Intesti-bacteriophage to study 219 patients suffering from either dysentery (84 children and 27 adults with *Shigellashiga* (now known as *Shigella dysenteriae*) or *flexneri*) orhemolytic intestinal disease caused by an unidentified bacterium (54 children and 54 adults).Most had previously been treated unsuccessfully in other ways, but other treatments were stopped during administration of the phage therapy. Adults were given 10 mL and children 2.5–5 mL orally with carbonated water once a day, before meals. Improvement was observed in 163 cases within 1–3 days. The results of this study and a number of others have been summarized in great detail by Chanishvili [21] in her extensive 2009 literature review of the early practical application of bacteriophage research, previously largely available only in Georgian.

There is an unknown, quite large total number of phages in the Eliava Intestiphage cocktail, which has continually been evolved to meet current needs since it was first developed by d’erelle at the Pasteur Institute. At least one proprietary mother phage stock has been maintained through the years for the phages targeting each genus of bacteria, and each of these is grown separately using a proprietary group of bacterial strains of that genus, which is updated regularly as needed to be able to better target new problem strains that have arisen. Each component thus produced for a new commercial batch is tested on each member of a separate continually-updated broad proprietary group of strains and remade if it does not adequately meet the established high host range for that genus. New phages are periodically added to improve the needed host range for this broadly-applicable commercial cocktail, which has been shown to have such high efficacy in a variety of situations, both as a probiotic and to treat a wide range of gut problems that are often intransigent to more narrowly targeted phage treatments and/or to antibiotic treatment. This challenges most current common regulatory practices in countries other than Georgia, where the carefully defined method of testing and regulation of Intestiphage takes this into consideration, with close cooperation between the Ministry of Health regulatory body and the production facilities. The procedure described above for preparing therapeutic bacteriophage is similar to the procedure described in a chapter on phage production by Felix d’Herelle. The original chapter has been translated into English by Sarah Kuhl and Hubert Mazure [22].

The Eliava Pyophage cocktail, for purulent infections involving *Streptococcus* sp., *Proteus* sp., *Escherichia coli*, *Pseudomonas aeruginosa* and *Staphylococcus aureus*, is the one other cocktail that has evolved in similar fashion over the years. It should be kept in mind that Intestiphage and Pyophage are generic names; other companies in both Georgia and Russia have been making and marketing their own versions for the last couple of decades which have been evolved from the same initial cocktails brought to what is now the Eliava Institute by d’Herelle and are regulated and regularly upgraded in similar fashion. These other versions can be expected to work better in some specific situations, worse in others, depending on their precise composition of phages and of the proprietary hosts that are used in their production and testing. It will be very interesting to also do metagenomic analyses of those other versions and see how their current composition compares, in reflection of this evolutionary process.

## 2. Materials and Methods

### 2.1. The Intesti Phage Cocktail

Commercial “Intesti bacteriophage”, which is used mainly to treat bacterial infections of the intestine, urinary tract and oral cavity in humans, was kindly provided by Nikoloz Nikolaishvili, director of Eliava Bio Preparations LLC at the George Elivia Institute, Tbilisi, Georgia. The current Eliava Intestibacteriophage contains sterile phage lysates active against *Shigella* (*flexneri*, *sonnei*, Newcastle), *Salmonella* (Paratyphi A, Paratyphi B, Typhumurium, Enteritidis, Cholerasuis, Oranienburg), *Escherichia coli*, *Proteus vulgaris and mirabilis*, *Stapylococcus aureus*, *Pseudomonas aeruginosa* and *Enterococcus*. Intestibacteriophage is used for treatment and prophylaxis of the following bacterial intestinal infections caused by the above mentioned microorganisms: dysentery, salmonellosis, dyspepsia, colitis, enterocolitis, and dysbacteriosis (bacterial overgrowth). Intestibacteriophage treatment per os (via oral route) is used from the first day of disease and is continued for 5–6 days. Intestibacteriophage can be used for prophylaxisin situations where there are large groups of people (for example military or schools), during seasonal peaks in order to reduce occurrence of intestinal infections. The phage preparation developed for therapeutic and prophylactic uses by G. Eliava Institute of Bacteriophages, Microbiology and Virology was awarded in 1978 Gold Medals at the Exhibitions of All-Union National Achievements in Science and Technology.

From the mode of preparation, it follows that the Intesti cocktail is a complex mixture of phages in different abundances, many of which may be closely related. This poses certain challenges both in the sequencing and assembly. Furthermore, different batches of the cocktail may not be identical. Our sample was manufactured in July 2013 and has the batch number M2-501.

### 2.2. Host-Amplified Samples

In addition to sequencing the complete cocktail as a metagenome, we also amplified the component phages on eight different hosts and isolated DNA from the resulting lysates, which are assumed to be enriched only in the phages capable of infecting the given host. Those samples are therefore reduced in complexity in comparison to the cocktail. The host strains used are part of an in-house Danish collection and listed in Table 1 (Results Section). For each host, 5 mL liquid LB were inoculated with 50 µL from an overnight culture and grown with shaking incubation at 37 °C. After 3 h the day culture was divided into two 2.5 mL samples, of which one was infected with 300 µL of the cocktail and incubated for another 4 h with shaking. When the infected sample had visibly cleared compared to the non-infected sample, indicating that host lysis had occurred, the lysate was filtered through 0.22 µm syringe filters and subsequently treated the same as the Intesti whole cocktail sample (see Sample Preparation). It should be noted that the bacterial host strains used to produce the cocktail in Georgia are proprietary and thus were not available to us in Denmark.

**Table 1:** List of the strains used to specifically amplify phages from the Intesti cocktail and the number of reads obtained in their sequencing. All strains were tested for susceptibility to the cocktail prior to selection.

Host Bacterial Strain	Number of Reads
*Escherichia coli* ATCC 25922	358,914
*Enterococcus faecalis* ATCC 29212	134,966
*Pseudomonasaeruginosa* 0407431-2	184,790
*Pseudomonasaeruginosa* PAO1_seq	265,772
*Proteus vulgaris* CCUG 36761 (ATCC 13315)	64,852
*Salmonella typhimurium* ATCC 14028	133,980
*Shigellaflexneri* iran_1s	225,664
*Shigellasonnei* iran_2s	401,722

### 2.3. Sample Preparation

All phage samples intended for sequencing were treated with 10 µL (20 units) of 2000 units/mL DNAse (New England BioLabs, Ipswich, MA, USA) per mL of phage lysate and 5 µL of 20mg/mL RNase (Invitrogen, Carlsbad, CA, USA) per mL of phage lysate to remove possible bacterial DNA leftovers. Subsequently, the samples were treated with 4µL of 20 mg/mL Proteinase K (Merck Milipore, Hellerup, Denmark) per mL of phage lysate to open phage capsids, followed by standard DNA extraction by spin column using the Phage DNA isolation kit by NorgenBiotek (Product #46700, Thorold, ON, Canada).

### 2.4. Sequencing and Genome Assembly

For each sample a DNA library was prepared from 10 ng of sample DNA using the Nextera XT Sequencing kit (Part #15031942, Illumina, San Diego, CA, USA) and sequencing was performed on the Illumina MiSeq system (Illumina, San Diego, CA, USA). The platform’s maximum read length was 251 bp corresponding to 251 cycles. The quality of the raw sequencing data was analyzed with the fastQC tool [23] and it was trimmed extensively using the PRINSEQ [24] tool (trimming parameters may be found in the Supplementary Table S1). Following quality trimming, the data were assembled into contigs using the genovo algorithm [25] for the whole cocktail and samples amplified on *E. coli*, *Enterococcus*, *P. aeruginosa* PAO1_seq, *Salmonella*, *Shigellaflexneri* and *Shigellasonnei* and the velvet [26] assembler for samples amplified on *P. aeruginosa* 0407431-2 and *Proteus*.

### 2.5. Construction of Phage Clusters

**Figure 1:**
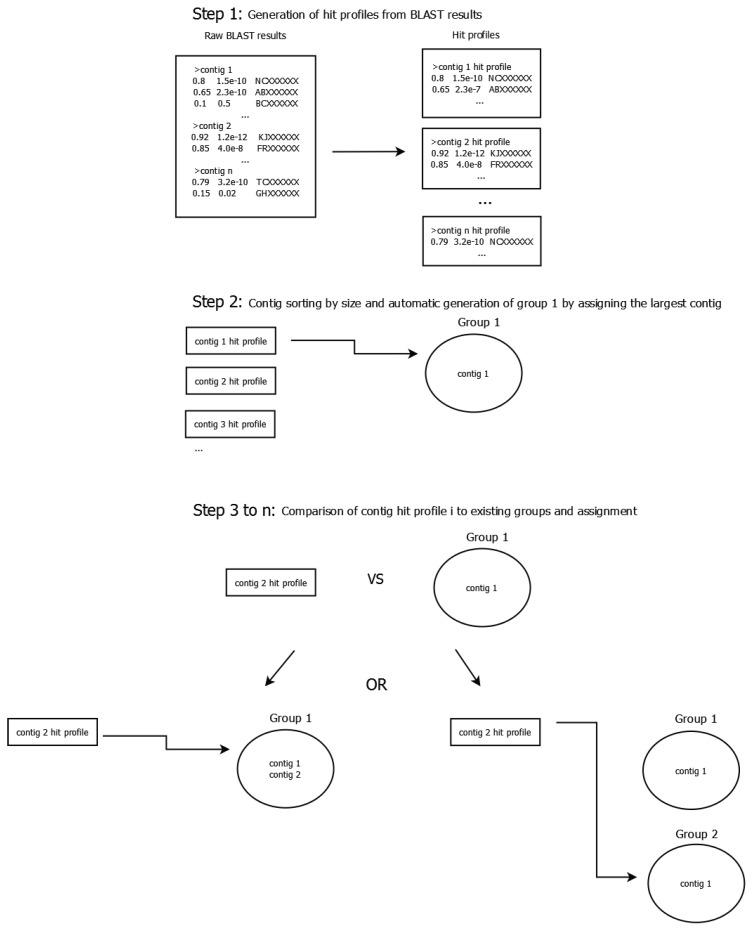
Schematic illustrating the contig grouping process. In a first step, a BLAST search against the non-redundant nucleotide collection is performed for all contigs. Afterwards, a hit profile is generated for each contig by applying a cutoff of 20% on the query coverage and 1 × 10^−10^ on the *E*-value to the raw BLAST results. During the second step contigs are sorted by size and the largest contig is automatically assigned to group 1. The third step consists of comparing the second-largest contig to all existing groups using the scoring system described in the text and either assigning the contig to the group with the lowest distance score or opening a new group if the lowest score is greater than 0.9. It is repeated until all contigs have been assigned (though some contigs may be the only member of their respective group).

Phage clusters were constructed by grouping contigs by their profiles of BLAST [27] hits to NCBI’s non-redundant nucleotide collection (October 2014). Those hit profiles were obtained by applying a quality cutoff on the query coverage of 20% and on the *E*-value of 1 × 10^−10^ to the raw BLAST results. Contigs were sorted by size and the largest was automatically assigned to the first contig group. Succeeding contigs either joined an existing group or initiated a new one depending on the distance score (see below) between the current contig’s hit profile and the group’s hit profile. The process is illustrated in Figure 1. Because of the high complexity of the cocktail, we find it useful to think of those drafts as representing clusters of related phages and they are henceforth referred to as clusters.

The distance score *S_d_* between two profiles was defined as the average distance of each hit in both profiles such that:

- If the hit is only present in one of the profiles, its distance is 1.0.
- If the hit is present in both profiles, the hit’s distance is the absolute value of the difference between the query coverage values, as defined below:

(1) $$S_{d}\left(profile_{l},profile_{k}\right)=\frac{\sum_{i=1}^{n}\{\begin{array}{c} abs\left(querycoverage_{hitiinprofilel}-querycoverage_{hitiinprofilek}\right)\\ 1.0;ifnothit_{i}\in profile_{l}\vee nothit_{i}\in profile_{k} \end{array}}{n}$$

where *n* is the unique number of hits in profiles *l* and *k*.

A contig group’s hit profile is the weighted average of the hit profiles of its member contigs and it was updated every time a contig joined the group. The query coverage, *i.e.*, to which extent a contig is covered by that particular hit was thereby used as a scaling property ranging between 0 and 1. The more of a contig is represented by the hit, the bigger the influence of that hit on the difference score. This was done to address the modular nature of phage genomes [28].Contigs that had database hits which were not shared by any other contigs were compared to known phages with regard to length, coverage of the contig by the reference and percent sequence identity, in order to establish whether they could be representing full phage genomes. Contig groups smaller than 5 kb in total size were excluded from further analysis. They represent less than 1% of the assembly size and mostly had hits to bacterial DNA, though upon further investigation many of those hits turned out to be confirmed or suspected prophage or mobile element regions.

We further employed BLAST to identify contig groups from different samples that are thought to originate from the same phage cluster. Contigs from the sample amplified on a *Proteus* host were compared to NCBI’s non-redundant nucleotide collection (October 2014) and after checking for sufficiently high depth of coverage those without hits were considered as belonging to novel *Proteus* phages.

### 2.6. Analysis of the Depth of Coverage

The average depth of coverage was calculated for each contig by mapping the reads that were previously used for assembly back to the contig. Following that, the average depth of coverage for each cluster was calculated from the depth of coverage of its member contigs. We herein incorporated contig length as a scaling factor in the calculation and thereby obtained the weighted arithmetic mean of the cluster’s depth of coverage and weighted standard deviation of the same as defined below.

Depth of coverage of contig *i*,

(2) $$x_{i}=\;\frac{N\times L}{w_{i}}$$

weighted mean depth of coverage of cluster *j*

(3) $${\bar{x}}_{j}=\;\frac{\sum_{i=1}^{n}w_{i}\times x_{i}}{\sum_{i=1}^{n}w_{i}}$$

and weighted standard deviation of the depth of coverage of cluster *j*

(4) $${\bar{\sigma }}_{j}=\;\sqrt{\frac{\sum_{i=1}^{n}w_{i}\times {\left(x_{i}-{\bar{x}}_{j}\right)}^{2}}{\sum_{i=1}^{n}w_{i}}}$$

as used in this study, where *N* = number of reads mapped to contig *i*, *L* = average read length, *x_i_* = depth of coverage of contig *i*, weight $w_{i}$ = length of contig *i* and *n* = the number of contigs in cluster *j*.

Mapping was performed using the Burrows-Wheeler Alignment tool (BWA) [29]. Prior to mapping, reads were quality trimmed (specifics may be found in Supplementary Table S1), however, duplicates were not removed as had been done for the assembly.

### 2.7. Gene Prediction and Functional Annotation

Putative genes were predicted in both grouped and un-grouped contigs. Nineteen near complete draft genomes were submitted to the annotation server RAST [30] for functional annotation. Additionally, gene calling was performed on all contigs using the GeneMarkS algorithm [31], followed by a BLAST search against NCBI’s non-redundant protein database to infer annotation from existing homologs and achieve an overview of the functions present in the phage cocktail. Annotation was hereby extracted from the top BLAST hit with the additional requirement that the match to this top hit had an *E*-value smaller than or equal to 1 × 10^−10^. The results of the two approaches were then compared. Two genes were considered to be the same if their start and end coordinates were less than 10% of the gene length apart and in frame of each other; that is, if the difference between the coordinates for the two genes was a multiple of three. The obtained annotation was subsequently text-mined for genes considered to be undesirable in phage therapy, such as bacterial virulence factors and genes related to lysogeny [32], as well as for genes speculated to enhance the phages’ efficacy. For this part, we chose to focus on methylase genes which have been discussed as a method to evade restriction by the bacterial host [33]. Furthermore, the complete assembly was scanned against a database of known genes for acquired antimicrobial resistance by using the ResFinder tool [34] and against a database of known virulence genes in *E. coli*, *Enterococcus* and *Staphylococcusaureus* using the VirulenceFinder tool [35]. No gene prediction and annotation was performed in the host-amplified samples.

### 2.8. Host Range Estimation

Lastly, in order to verify the cocktail’s capability to cause lysis of the specified pathogens, five to ten strains were selected for each pathogen and tested for susceptibility towards the phage cocktail by streaking the bacteria onto an agar plate perpendicular to a streak of phage solution. The selection was oriented towards maximum diversity, including strains from different geographical origins and different host reservoirs. For the pathogens only listed at genus level, different species were tested. The strains and test results can be found in Supplementary Table S2. If lysis occurred in the intersection zone, the bacterial strain was registered as being susceptible to the cocktail. Ambiguous results were repeated in triplicate.

## 3. Results

### 3.1. Sequencing Statistics

After quality trimming the sequencing of the full Intesti cocktail resulted in 440,392 reads with an average read length of 174.9 bp. *De novo* assembly yielded 420 contigs ranging in size from 500 to 134,226 bp and a total assembly size of 2041 kb.

In the host-amplified samples, the sequencing depth varied between the different samples. This is indicated by the differing number of reads, see Table 1. Some of the reasons for this could be a variation in the input DNA concentration, as well as amplification bias during library preparation and during the sequencing process.

**Table 2:** Overview of selected characteristics of the phage clusters identified in the Intesti sample. If known, the family, subfamily and genus of the closest database reference as specified by the ICTV are given. In some cases, the closest reference phage has not been incorporated into the phage taxonomy yet but other references have. For those, both the closest reference and the closest reference within the taxonomy scheme are given. The genus “rv5-like virus” has been proposed by several authors [36,37], but is not confirmed in the current (2014) ICTV release. Remark that Bacteriophage G1 is annotated as a *Staphylococcus* phage.

Phage Cluster	Cluster Size in bp	Reference Accession	Average Coverage of Phage Cluster	Average Percent Identity	Reference Phage Description Line	Phage Family	Subfamily	Genus	Size RatioCluster/Reference
D1	142,025	KC012913.1	99.97	99.80	*Staphylococcus phage Team1*, complete genome	*Myoviridae*			1.01
		AY954969.1	97.98	99.74	*Bacteriophage G1*, complete genome *		*Spounavirinae*	*Twortlikevirus*	1.02
D2	76,960	JX415536.1	87.89	87.60	*Escherichia phage KBNP135*, complete genome	*Podoviridae*			1.00
D3	87,828	KC862301.1	98.97	96.16	*Pseudomonas phage PAK_P5*, complete genome	*Myoviridae*			1.00
D4	69,023	KF562340.1	87.20	94.02	*Escherichia phage vB_EcoP_PhAPEC7*, complete genome	*Podoviridae*			0.96
D5	150,530	FR775895.2	92.41	98.16	*Enterobacteria phage phi92*, complete genome	*Myoviridae*			1.01
D6	81,563	AB609718.1	35.55	77.46	*Enterococcus phage phiEF24C-P2* , complete genome	*Myoviridae*			0.57
D7	58,193	KJ094032.2	77.23	88.35	*Enterococcus phage VD13*, complete genome	*Siphoviridae*	-	*Sap6likevirus*	1.06
D8	50,277	HM035024.1	98.16	90.67	*Shigella phage Shfl1*, complete genome	*Siphoviridae*	-	*Tunalikevirus*	0.99
D9	39,912	EU734172.1	88.25	93.45	*Enterobacteria phage EcoDS1*, complete genome	*Podoviridae*			1.02
D10	145,982	KJ190158.1	93.95	93.00	*Escherichia phage vB_EcoM_FFH2*, complete genome	*Myoviridae*			1.05
		DQ832317.1	93.72	92.62	*Escherichia coli bacteriophage* *rv5*, complete sequence		-	“rv5-like virus” *	1.06
D11	61,791	JX094499.1	96.33	92.95	*Enterobacteria phage Chi*, complete genome	*Siphoviridae*			1.04
		KC139512.1	95.15	93.86	*Salmonella phage FSL SP-088*, complete genome		-	*Chilikevirus*	1.04
D12	60,451	KJ010489.1	54.57	87.35	*Enterococcus phage IME-EFm1*, complete genome	*Siphoviridae*			1.42
D13	188,630	GU070616.1	88.67	94.90	*Salmonella phage PVP-SE1*, complete genome	*Myoviridae*		“rv5-like virus” *	1.29
D14	133,015	JX128259.1	94.55	96.24	*Escherichia phage ECML-134*, complete genome	*Myoviridae*			0.80
		DQ904452.1	93.42	96.00	*Bacteriophage RB32*, complete genome		*Tevenvirinae*	*T4likevirus*	0.80
D15	43,967	GQ468526.1	87.06	91.27	*Enterobacteria phage 285P*, complete genome	*Podoviridae*			1.12
		FJ194439.1	87.13	90.61	*Kluyvera phage Kvp1*, complete sequence		*Autographivirinae*	*T7likevirus*	1.11
D16	46,882	KM233151.1	93.68	91.47	*Enterobacteria phage EK99P-1*, complete genome	*Siphoviridae*			1.06
		JX865427.2	91.64	91.03	*Enterobacteria phage JL1*, complete genome			*Hk578likevirus*	1.08
D17	41,098	AY370674.1	88.68	94.28	*Enterobacteria phage K1-5*, complete genome	*Podoviridae*	*Autographivirinae*	*Sp6likevirus*	0.93
D18	41,016	HE775250.1	94.95	91.57	*Salmonella phage vB_SenS-Ent1* complete genome	*Siphoviridae*			0.97
		JX202565.1	92.76	91.41	*Salmonella phage wksl3*, complete genome			*Jerseylikevirus*	0.96
F1	13,855	HG518155.1	99.97	99.02	*Pseudomonas phage TL* complete genome	*Podoviridae*			0.30
		AM910650.1	91.92	97.11	*Pseudomonas phage LUZ24*, complete genome		*-*	*Luz24likevirus*	0.30
F2	11,476	EU877232.1	99.94	91.42	*Enterobacteria phage WV8*, complete sequence	*Myoviridae*	*-*	*Felixounalikevirus*	0.13
F3	5706	HQ665011.1	83.42	86.09	*Escherichia phage bV_EcoS_AKFV33*, complete genome	*Siphoviridae*			0.05
		AY543070.1	82.09	87.59	*Bacteriophage T5*, complete genome		*-*	*T5likevirus*	0.05
F4	2624	EF437941.1	98.59	97.76	*Enterobacteria phage Phi1*, complete genome	*Myoviridae*	*Tevenvirinae*	*T4likevirus*	0.02
Proteus phage	104,213	-	-	-	-	*Siphoviridae*			-

### 3.2. Recovered Phage Clusters

Within the cocktail, 22 phage clusters were recovered by grouping using BLAST hit profiles (see Materials and Methods); plus one novel *Proteus* phage cluster was cluster identified by comparing contigs without hits between the Intesti sample and the *Proteus* host-amplified sample. All clusters are listed in Table 2. They are denoted by a capital D and numbered, except for four smaller clusters under 30 kb in size, which are regarded as containing fragments of phages and therefore denoted by capital F instead. The reason those four clusters are thought to be fragments is that they are small compared the known phages they resemble most, while the other clusters are of similar or greater size than their BLAST hit. It is acceptable for a cluster to be of greater size since the cluster size is cumulative of all member contigs and there can be several variant phages. Overall, clusters ranged in size from 13.4 to 212 kb and were composed of between one and 56 contigs. Seventy contigs, which together make up 217 kb of sequence or 10.6% of the total assembly size, had no significant hits to NCBI’s nr nucleotide database. They could therefore not be assigned to a cluster. A list of clusters recovered in the host-amplified samples may be seen in Supplementary TableS3.

#### 3.2.1. Similarity to Known Phages

The most significant BLAST hits used to form the phage clusters were used to examine which known phages a cluster seems to be related to. In Table 2 the reference phage with the highest identity is listed for each cluster, together with the family and, if given, subfamily and genus of that phage according to the ICTV. In cases where there is no taxonomical data available for the closest match but for another match, this reference phage is also listed (compare D14, D15, D16, D18, F1 and F3). Based on the phage family of their closest references, we inferred the potential family association of the clusters. A BLAST search of the predicted tail fiber, DNA polymerase and capsid genes of the *Proteus* phage revealed them to be most similar to those of *Siphoviridae*. We therefore predict the *Proteus* phage cluster to belong to the *Siphoviridae* and count the reads mapped to it into that family. While larger than most studied *Siphoviridae* (which are around 50 kb), the 104 kb Proteus cluster is still smaller than the genomes of the T5 genus of phages. The depth of coverage is quite even along the two contigs in this cluster, so it seems unlikely that the length has been artificially increased through collapsing multiple phages into the cluster.

The clusters could be divided into three groups based on their similarity to their reference phages: Clusters with several highly similar references (query coverage and percent identity >90%), cluster with medium similar references (query coverage and percent identity between 90% and 70%) and clusters that were very distant from all publically available phage sequences. The clusters with several highly similar references are D1, D3, D8, D10, D11, D14, D16, D18, F1, F2 and F4. Specifically for D1 and D3, the resemblance to their closest database reference was very pronounced. We therefore conclude that we have identified phages that appear to be of the same phage species as *Staphylococcus* phage Team1 (KC012913.1) and *Pseudomonas* phage PAK_P5 (KC862301.1), respectively, in the Intesti phage cocktail. The other eight clusters in these groups can also be viewed as fairly close relatives of the clusters described by their reference phages. The second group of clusters, with a slightly lower but still apparent similarity to their references, was D2, D4, D5, D7, D9, D13, D15, D17 and F3. These clusters contain parts that differed from their references, either because they were acquired from other phage species or because they are novel. In contrast, the references for the clusters D6 and D12 were quite distant, as can be seen by the low query coverage. This means that large parts of those two clusters are novel.

Regarding the inferred taxonomy of the clusters, we were able to assign 13 of the clusters to a suspected genus. Of those, four were assigned to the *Myoviridae* genera *Twortlikevirus*, *T4likevirus* (two clusters) and *Felixounalikevirus*. A further six clusters were assigned to the *Siphoviridae* genera *Sap6likevirus*, *Tunalikevirus*, *Chilikevirus*, *Hk578likevirus*, *Jerseylikevirus* and *T5likevirus*. Finally, three clusters were assigned to the *Podoviridae* genera *T7likevirus*, *Sp6likevirus* and *Luz24likevirus*. Two more clusters had reference phages that have been proposed for the new *Myoviridae* genus *rv5-like virus*, however this genus remains unconfirmed in the 2014 ICTV release. Another six clusters have reference phages, which have not been placed in the official taxonomy yet. Furthermore, the cluster D6 and the Proteus phage cluster may represent entirely new taxa.

#### 3.2.2. Depth of Coverage in the Intesti Clusters

**Figure 2:**
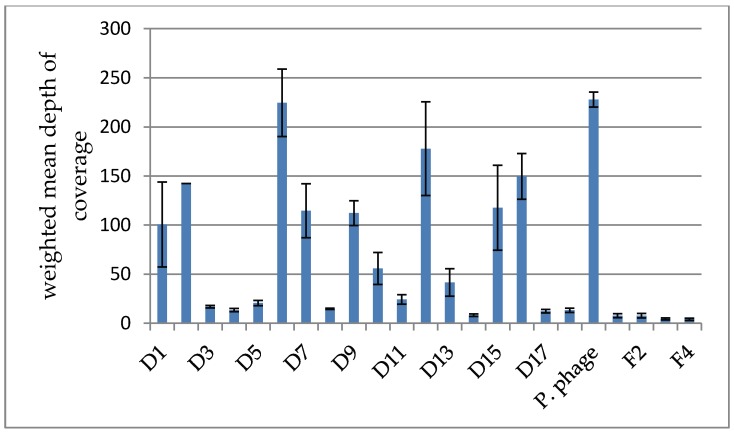
Comparison of the weighted mean of the depth of coverage between clusters in the Intesti sample. The weighted standard deviation is depicted as error bars. Note that cluster D2 is composed of only one contig and the standard deviation is therefore not applicable. It can be seen that the depth varies greatly between clusters, reflecting the different abundances of the represented phage types in the cocktail.

It was found that the weighted average depth of coverage varied considerably between clusters, indicating a different abundance of those clusters within the cocktail (compare Figure 2). D6 and D12 as well as the *Proteus* phage cluster were found to be particularly abundant with an average depth of coverage greater than 150×. In contrast, the clusters D3, D4, D5, D8, D11, D14, D17 and D18 had a very low average depth of coverage of 10× or less.

Furthermore, we observed that many clusters exhibited some degree of variation in the depth of coverage between their member contigs, evident by the weighted standard deviation, which is shown as error bars in Figure 2. Upon inspection, we found that this was generally caused by a few contigs with a very different depth from the rest (compare supplementary Figure S1). We reason that those contigs can be explained by one of the following two scenarios.

In a sufficiently closely related cluster, most of the common genome will assemble into a few long contigs with a high depth of coverage. The parts that differ between phages in the cluster, however, assemble into contigs that have a much lower depth. In that case, the depth of coverage is proportional to how common the module represented by that contig is within the cluster. Low coverage contigs may also be variants of the more common sequence contained in the high coverage contigs. Contrary to that, in a less closely related cluster, the parts of the phage genome that are shared can assemble into a few chimeric contigs instead of being placed in their respective genomes, causing those contigs to have excess coverage compared to the rest.

Furthermore, we looked at the abundances of the phage families by summing the reads mapped to all clusters inferred to be *Myoviridae*, the same for *Podoviridae* and *Siphoviridae*. Reads mapping to contigs not assigned to a cluster are counted as unknown family. Doing that, we observed 35% *Myoviridae*, 15% *Podoviridae* and 32% *Siphoviridae* in the reads. On top of that, 18% of the total reads are of unknown family. Observe that those fractions refer to reads that are quality trimmed but not redundancy reduced. When doing the same procedure with redundancy reduced reads, the fractions change to 41% *Myoviridae*, 16% *Podoviridae*, 29% *Siphoviridae* and 14% unknown family.

#### 3.2.3. Depth of Coverage in the Host-Amplified Samples and Comparison of Phage Clusters between Samples

After performing contig grouping in the host-amplified samples, we examined each clusters’ highest scoring hits to phage in the non-redundant nucleotide collection and compared to the highest scoring hits in the Intesti clusters. Based on that, we identified clusters across samples that appeared to be synonymous. Using the ratio of the depth of coverage in the host amplified sample to the depth of coverage in the non-amplified Intesti sample, we were able to identify the infecting clusters since those experienced a great rise in coverage, up to 1000-fold (compare Table 3). All of the samples show significant amplification in only a few of the clusters. D14 was able to infect *E. coli* as well as both *Shigella* species, which is concurrent with the notion that those two species are closely related [38]. The two *Shigella* species tested were found to be susceptible to the same two clusters D14 and D15. Both of those appeared to be relatives of *Escherichia* or *Enterobacteria* phages. The *Enterococcus* and *Salmonella* samples shared two infecting clusters, namely D18 and F2. The authors are doubtful of the truth of this result, as *Enterococcus* is Gram positive and *Salmonella* Gram negative. It has therefore been removed.

**Table 3:** Depth of coverage ratio of host-amplified samples to the Intesti sample. Combinations with a ratio greater than 1.0 are indicated by green background coloring. Those are thought to be the infecting clusters, as they are more abundant in the host-amplified sample than in the original one. In the last line is shown a phage cluster, which has not even been considered in the initial contig grouping of the Intesti sample because of its small size of only 1346bp and low depth of coverage of only 2×. It has, however, been greatly amplified on *P. aeruginosa* strain PAO1.Results regarding the amplification on Salmonella were inconclusive and therefore removed (see text).

Cluster	*E. coli*	*Enterococcus*	*P. aeruginosa* PAO1	*P. aeruginosa* PA0407	*Shigella flexneri*	*Shigella sonnei*	*Proteus*
D1	0.03	0.00	0.00	0.00	0.02	0.00	0.00
D2	0.02	0.00	0.00	0.00	0.02	0.02	0.00
D3	0.30	0.00	0.00	22.29	0.10	0.00	0.00
D4	0.09	0.00	0.00	0.00	0.00	0.00	0.00
D5	0.11	0.00	0.00	0.00	0.00	0.00	0.00
D6	0.06	0.00	0.02	0.00	0.01	0.01	0.00
D7	0.05	2.57	0.00	0.00	0.02	0.00	0.00
D8	0.00	0.00	0.00	0.00	0.00	0.00	0.00
D9	0.04	0.00	0.00	0.00	0.02	0.00	0.00
D10	0.08	0.00	0.06	0.00	0.02	0.00	0.00
D11	0.13	0.13	0.00	0.00	0.00	0.00	0.00
D12	0.04	0.00	0.01	0.00	0.02	0.00	0.00
D13	0.05	0.05	0.00	0.00	0.04	0.00	0.00
D14	4.74	0.00	0.00	0.00	2.82	2.06	0.00
D15	0.04	0.00	0.00	0.00	4.97	9.84	0.00
D16	0.04	0.00	0.00	0.00	0.02	0.02	0.00
D17	47.17	0.00	0.00	0.00	0.00	0.00	10.01
D18	0.37	-	0.00	0.00	0.00	0.00	0.00
F1	0.00	0.00	1.47	0.00	0.00	0.00	0.00
F2	0.00	-	0.00	0.00	0.00	0.00	0.00
F3	0.00	0.00	0.00	0.00	0.00	0.00	0.00
F4	0.00	0.00	0.00	0.00	0.00	0.00	0.00
*Proteus*	0.04	0.00	0.02	0.00	0.00	0.01	0.12
*	0.00	0.00	1044.20	0.00	0.00	0.00	0.00

Note: The cluster marked by an asterisk (*) exists in the Intesti sample but has not been named due to its small size and low depth (see table header).

BLAST-based comparison of those infecting clusters confirmed that they had a highly similar sequence content to the clusters in the unamplified Intesti sample. With the exception of two clusters amplified on *P. aeruginosa* PAO1, all others clusters were also of similar length when compared between samples. F1, which is a fragment cluster in the Intesti sample, probably due to low abundance of those phages in the cocktail, nearly doubled in size to 22,920 bp on the PAO1 sample. Despite this, about half of the sequence content of the F1 cluster in the Intesti sample is not represented in the F1 cluster in the PAO1 sample. This indicates that F1 contains at least two distinct phages, only one of which was amplified on PAO1, and this amplification enabled us to recover more of the sequence of that phage. Furthermore, a new cluster of length 45,478 bp appeared in the PAO1 sample. There is evidence of this cluster in the Intesti sample but was not treated as such due to its very small size of 2392 bp and low depth of coverage of 1.78×. Those results gave us more confidence that the clusters defined by us are meaningful within the context of the cocktail.

Certain samples as e.g., the one amplified on *E. coli* also contained many different clusters in low abundance. We believe that those phages are un-amplified phages carried over from the cocktail when the host culture was infected. This is backed up by the fact that those clusters are synonymous to Intesti clusters with a high depth of coverage and they are predominantly observed on those host-amplified samples that featured a high read-count. Additionally, we found no indication that the phage cluster we think to be a cluster of *Proteus* phages is capable of infecting the *Proteus vulgaris* strain we used for amplification.

#### 3.2.4. Gene Prediction and Functional Annotation in the Intesti Clusters

Gene prediction via GeneMark S on all contigs yielded a total of 3013 genes, 2577 of which were predicted on the contigs that were assigned to a phage cluster and 258 of which were predicted on unassigned contigs. 2864 genes (95%) had hits to NCBI’s non-redundant protein database and annotation was retrieved from the top hits. It was however found to be of limited usefulness since it is not standardized or focused on molecular function and often consists of unspecific terms such as “hypothetical protein” or terms that only carry meaning within the genome they were originally annotated in like “ORF3245”.

The RAST service, which was only used on the phage clusters, predicted 2408 genes. RAST uses homology to genes in internal databases to retrieve annotation for the genes it calls. If this fails, the annotation line “hypothetical protein” is given, though it can also be obtained by homology to a gene already annotated in that way. A total of 893 genes (37%) carry the “hypothetical protein” annotation. The overlap between genes predicted by RAST and GeneMarkS was 2230 genes.

Phages with the ability to integrate into the host’s genome are known to often carry genes that increase their host’s fitness, among those resistance genes and virulence factors. For that reason, integrase genes are generally regarded as undesirable in a phage therapy context [3]. The full assembly of the cocktail’s metagenome was scanned against databases of resistance genes and virulence genes using the ResFinder [34] and VirulenceFinder [35] tools. Neither scan detected the presence of any known antimicrobial resistance genes or bacterial virulence factors for *E. coli*, *Enterococcus* or *Staphylococcus*. Text mining the annotation for the terms “resistance” and “virulence” returned seven genes in the RAST annotation, which are listed in Table 4. All but one of those genes were also predicted by GeneMarkS, but differently annotated through BLAST. None of these genes, however, seemed to be related to antibiotic resistance. A literature search determined that the identified resistance genes were related to antiseptic resistance, which is not regarded as problematic as antibiotic resistance [39] but also not desirable, especially in relation to the treatment of pathogens. On the other hand, antiseptics like acridine and acriflavine have been shown to inhibit phage activity [40,41], so the presence of resistance genes against those agents might be a tradeoff between achieving the highest possible safety and retaining efficacy of the phage cocktail. Furthermore, one of the most thoroughly lytic phages T4 can become resistant to inhibition of replication by acridine and acriflavine [42].The two proteins annotated as “Phage virulence-associated protein” have tail proteins among their closest BLAST hit, so it can be assumed that the term refers to virulence of the phage towards its host and not to bacterial virulence factors.

**Table 4:** List of genes potentially relevant for efficacy, found by text mining annotation results. The annotation column details whether the gene was found in the annotation provided by RAST, by BLAST or both. If only one is named the other method either did not predict the gene or annotated it differently. Top BLAST hit, query coverage as given by BLAST and percent identity as given by BLAST are only filled out if applicable. Most genes which were picked up for their RAST annotation still have a BLAST hit description line, query coverage and percent identity values because that gene was also called by GeneMarkS. In any case, the last two columns apply to the BLAST hit, but not necessarily to the hit in the RAST databases. The acridine resistance gene evidenced in D14 was not called by GeneMarkS. If the gene was picked up for its BLAST annotation column 2 and 5 are identical.

Text Mining Term	Description Line	Part of Cluster	Annotation by	Top BLAST Hit Description Line	Query Coverage	Percent Positives
“virulence”	Phage virulence-associated protein	D1	RAST	ORF002 (Staphylococcus phage G1)	100%	100%
	Phage virulence-associated protein	D6	RAST	putative adsorption associated tail protein (Enterococcus phage phiEF24C)	100%	95%
“resistance”	Acridine resistance	D14	RAST	-	-	-
	Acriflavin resistance protein	D3	RAST	hypothetical protein PAK_P500103 (Pseudomonas phage PAK_P5)	100%	100%
	Tellurium resistance protein TerD	D5	RAST	Phi92_gp172 (Enterobacteria phage phi92)	100%	100%
	Tellurium resistance protein TerD	D5	RAST	Phi92_gp173 (Enterobacteria phage phi92)	100%	100%
	Tellurite resistance protein	D5	RAST	Phi92_gp178 (Enterobacteria phage phi92)	100%	100%
“methyltransferase” or “methylase”	DNA methylase	D7	RAST/BLAST	See “Description line”	100%	99%
	DNA N-6-adenine-methyltransferase	D8	RAST/BLAST	See “Description line”	94%	90%
	putative site specific DNA methylase	D8	BLAST	See “Description line“	100%	99%
	DNA methyltransferase	D13	RAST/BLAST	See “Description line”	100%	99%
	putative DNA N-6-adenine methyltransferase	D10	RAST/BLAST	See “Description line”	100%	99%
	Dam methylase	D8	BLAST	See “Description line”	100%	100%
	putative DNA adenine methylase	D11	BLAST	See “Description line”	100%	100%
	putative DNA methyltransferase	unassigned	BLAST	See “Description line”	100%	100%
	DNA adenine methyltransferase	D14	BLAST	See “Description line”	100%	99%
	putative DNA adenine methylase	D11	RAST/BLAST	See “Description line”	100%	97%
	dCMPhydroxymethylase	D14	RAST/BLAST	See “Description line”	100%	100%
	putative adenine methyltransferase	D10	RAST/BLAST	See “Description line”	100%	98%
	DNA-cytosine methyltransferase	D5	RAST	Phi92_gp043 (Enterobacteria phage phi92)	100%	99%
	Adenine-specific methyltransferase	D5	RAST	Phi92_gp155 (Enterobacteria phage phi92)	100%	99%
“integrase”	Phage integrase	D2	RAST/BLAST	putative integrase (Escherichia phage KBNP1711)	100%	98%
	Phage integrase	D4	RAST/BLAST	integrase (Enterobacter phage IME11)	100%	99%

In addition to that, both annotation methods found two genes described as integrases in the clusters D2 and D4. The D2 integrase had a sequencing coverage of 110×, while the D4 integrase had a sequencing coverage of 11×. Both are congruent with to the coverage of the contigs they are placed in. Furthermore, both genes showed high similarity to known integrase genes (see Table 4). However, no statement can be made about the lysogenic or lytic nature of D2 and D4 phages since the integrity of the lysogeny module was not tested in the lab.

Lastly, 10 genes described as “methyl-transferase” or “methylase” were found in RAST’s annotation and 13 in the BLAST based annotation. We speculate that those genes may have a positive influence on efficacy as they can enable the phage to evade restriction-modification based defense systems as was detailed in a review by Samson *et al.* [33].

#### 3.2.5. Evaluation of Sequencing Depth of the Cocktail

**Figure 3:**
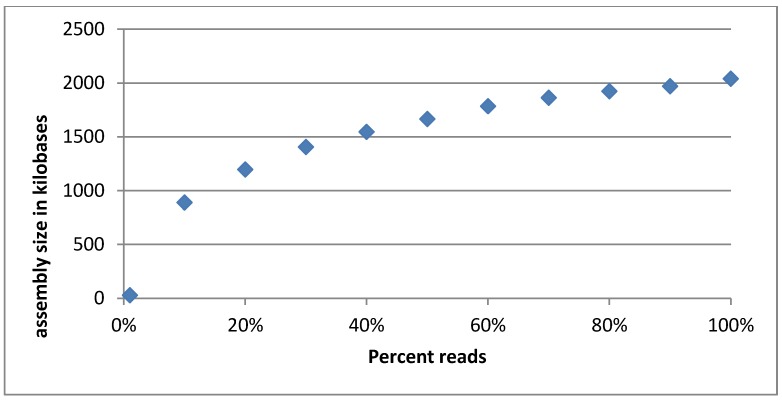
Rarefaction curve of the Intesti sequencing sample. The curve appears to flatten out as the percentage of reads used increases, indicating that the total assembly size is converging. This means that the most common phages are well represented in the sequencing reads. Phages that are in low abundance may not be adequately covered though.

A rarefaction curve was made by assembling discreet fractions of the quality trimmed reads and plotting the total assembly size *vs.* the fraction of reads used. The reasoning behind this was that if the phage cocktail has been sequenced sufficiently deeply, the assembly size will converge as more reads will add depth to the existing contigs instead of creating new ones. This behavior was indeed observed (compare Figure 3). It can be seen that the rarefaction curve is not completely flattened out, indicating that there may be rare phages not represented in the reads. Still, we reason that while the sample is not sequenced to its entire diversity we have succeeded in covering the majority of the phages present. Furthermore, when re-mapping reads to the finished assembly, 425,960 (97%) of the 440,392 reads map properly.

### 3.3. Host Range Estimation

In a small scale *in vitro* experiment we found the host range of the cocktail to be largely consistent with the specification given by the producer. Five to ten strains were tested for each pathogen listed on the package. The exact number of strains tested and the fraction of strains found susceptible are given in Table 5. The streaking tests confirmed that the cocktail was in principle able to cause lysis of strains of all seven pathogens specified by the producer, albeit with differing specificity for the different pathogens. The apparent low efficiency in lysis of *Staphylococcus* is due to the fact that only five of the ten tested isolates were *S. aureus*, of which all but one were susceptible. This can be seen in Supplementary Table S2, which also contains a complete list of the specific strains tested.

**Table 5:** Fraction of the strains found to be susceptible for each pathogen tested. Observe that this is only a small-scale experiment. All strains are part of an in-house collection.

Pathogen	Susceptible Strains
*Salmonella Enterica*	10/10
*Staphylococcus*	5/10
*Shigella*	5/5
*Pseudomonas Aeruginosa*	5/7
*E. coli*	2/6
*Proteus*	3/5
*Enterococcus*	2/5

## 4. Discussion

### 4.1. Completeness and Accuracy of the Analysis

The rarefaction curve showed that the phages that are numerically in the majority appear to be represented well in our data. However, there are indications that we have not seen the full diversity of the batch of Intesti we analyzed. A phage cluster amplified on PAO1was barely even present in the sequencing data of the cocktail, confirming that we potentially missed low abundance phages. It is not clear which impact the abundance of a particular phage or phage cluster has on its efficacy in the host, since specific amplification upon encountering the host is an important factor in therapeutic applications.

It is the authors’ understanding that the library preparation we used favors dsDNA and the vast majority of phages known today are indeed tailed dsDNA phages [4]. Nevertheless, we cannot exclude the possibility that the cocktail contained ssDNA phages, especially since we introduced a 5 kb size cutoff for contig groups. It is the authors’ experience, that contig groups smaller than that may not be true clusters but rather shared modules. At a size smaller than 5 kb it is further difficult to obtain an unambiguous attribution to a certain phage species or cluster of species due to the aforementioned shared modules.

Intriguingly, the three clusters that contain the most common phages in the cocktail, namely D6, D12 and the presumed *Proteus* phage cluster, are also those we know the least about, as they are the ones most different from previously studied phages. For the presumed *Proteus* phage, it is not even sure whether the two contigs form a single cluster, though each by itself is also very abundant (compare depth of coverage and its standard deviation for the *Proteus* phage, Figure 2). We have predicted the phages to belong to the *Siphoviridae* based on tail fibers, but it is not known what their hosts are.

There is a possibility that some of the phage components in the cocktail derive from induction of prophages in the propagating strains, which may explain the comparatively high prevalence of *Siphoviridae* in the Intesti cocktail as well as the presence of lysogeny-related genes. This hypothesis could not be tested since the propagating strains are proprietary and therefore not available.

### 4.2. Concerning the Synonymous Clusters and Amplification by Bacterial Hosts

It should be remarked that while the clusters infecting each host could be identified, it is not possible to say whether or not all phages in a given cluster are causing infection. In the case of cluster F1, of which only about half were amplified, the distinction was clear.

As was the case in the unamplified cocktail, the depth of coverage varied between contigs belonging to the same phage cluster in the host-amplified samples. This could signify a bias for amplification of only certain parts of the cluster. On the other hand, chimeric *vs.* non-chimeric contigs can also cause a variation in depth within a cluster (see Section 3.2.2).

Further, it turned out that the phage cluster we presumed to be *Proteus* specific, because of its presence in the Proteus amplified sample and the fact that it did not have any hits to the nr nucleotide database, did not actually cause infection in the *Proteus vulgaris* used in this study. It is therefore unclear what kind of phage those two contigs represent and whether they should be clustered or separate. The only evidence we have is that both of them have high depth of coverage values, which are very similar to each other.

### 4.3. Comparison to Other Phage Cocktail Studies Employing Metagenomics

McCallin *et al.* published a metagenomic analysis of a Russian phage cocktail intended for treatment of *Escherichia coli*/*Proteus* infections in 2013. Their methodology was somewhat different and more extensive on the experimental side. Our study had its focus on bioinformatics and specifically sequence analysis tools. These kinds of analyses are cheap and fast compared to traditional lab techniques which is why we wished to test their suitability for phage cocktail analysis. Naturally, they do not replace experimental evidence, however we think that by sequencing first and employing bioinformatics prior to further lab work, we are able to gain insight and can design lab experiments more efficiently. This will save time and money, especially as more tools are being developed and databases grow more extensive.

In concordance with the results of McCallin *et al.*, we also observed a great complexity within the cocktail we analyzed. McCallin *et al.* found primarily *Myoviridae* (34%) and *Podoviridae* (24%) in their cocktail. In comparison to that, the Intesti cocktail is also mainly composed of *Myoviridae* (35%), but the second most abundant family was *Siphoviridae*, which were almost as abundant (32%).The cocktail analyzed by McCallin *et al.* is, however, of very different scope, targeting solely *E. coli* and *Proteus*, while the Intesti cocktail we analyzed targets a more broad spectrum of enteric bacteria.

In the *Escherichia coli*/*Proteus* targeting cocktail, McCallin *et al.* identified phages of the *Myoviridae* subfamily *Tevenvirinae* and the genus *Felixounalikevirus*, plus phages of the proposed genus of rv5-like virus, as well as the *Podoviridae* genera *T7likevirus*, *SP6likevirus* and *N4likevirus*. The Intesti cocktail also contained clusters related to those two *Myoviridae* genera and subfamily and the *Podoviridae* genera *T7likevirus* and *SP6likevirus*. The Intesti cocktail appears to have a greater diversity of component phages compared to the Russian cocktail, which is in accord with its broader spectrum of application. As the sequencing data produced in the study of McCallin *et al.* is not publically available, the authors were unable to directly compare the phage clusters identified in the Intesti cocktail to the phages identified in the Russian cocktail.

Neither study identified undesirable genes within the cocktail, but this is not a guarantee for safety since the databases are not exhaustive. The two genes showing homology to integrases warrant further investigation.

When McCallin *et al.* classified their redundancy removed reads with MEGAN, they observed 23% of reads without hits. In comparison, 25% of the redundancy reduced reads in our sample mapped to contigs that could not be assigned, *i.e.*, had no significant BLAST hits. However, McCallin *et al.* compared their reads to the non-redundant protein collection and employed blastx, which has a higher sensitivity. Therefore, the numbers cannot be directly compared between the two studies. Furthermore, when looking at assembled contigs the total size of the contigs which had no database hits, including the putative Proteus phage, was only 16% of the total assembly size, though many of the clusters with known relatives appeared to have novel parts, as evidenced by the fact that their coverage by their database references is not complete (compare Table 2).

Lastly, the metagenomics approach differed between our study and that of the Russian phage cocktail in that we focused on assembling first and subsequently characterizing the contigs we had obtained, while McCallin *et al.* did more characterization work on the read data and with mapping. The main reason we chose direct *de novo* assembly of the full sample is that we were concerned about creating an artificial separation of the data by relying on mapping, especially since at least some phages are known to be modular and to frequently switch modules, as illustrated for *Staphylococcus* phages by Deghorain *et al.* [43]. Essentially, the focus of our study was on discovery.

### 4.4. Future Perspectives

One of the purposes of this study was to explore which types of sequence-based analysis are suitable for phage cocktails and whether their results are useful. We hope to ignite discussion on how the analysis of complex phage products can be done in the future.

## 5. Conclusions

The aim of this study was to identify and analyze the major components of the Intesti phage cocktail. Returning to the question posed in the title, we conclude that a great amount of information can be gained from examining a phage cocktail directly by metagenomic analysis, by relying on databases and bioinformatics tools, though careful interpretation is crucial and not always straight forward. Furthermore, we show that the kind of information presented in this article can be gained without the need to separate and amplify individual phages prior to sequencing, which may not always be possible especially when propagating strains are unavailable or unknown. As databases grow more extensive with sequencing projects on the rise and more tools get developed, we expect that the kind of bioinformatics analysis we employed in this study will grow more powerful and accurate.

## Supplementary Files

Supplementary File 1
