# Supplementary material for: What Can We Learn from a Metagenomic Analysis of a Georgian Bacteriophage Cocktail?
Source: Viruses. 2015 Dec 12;7(12):6570–89. doi: 10.3390/v7122958 (PMC4690881; doi:10.3390/v7122958)
Supplement: Supplementary File 1 [file viruses-07-02958-s001.pdf]

Supplementary Materials: What Can We Learn from a Metagenomic Analysis of a Georgian Bacteriophage Cocktail?

Henrike Zschach, Katrine G. Joensen, Barbara Lindhard, Ole Lund, Marina Goderdzishvili, Irina Chkonia, Guliko Jgenti, Nino Kvatadze, Zemphira Alavidze, Elizabeth M. Kutter, Henrik Hasman and Mette V. Larsen

Table S1. Overview of the trimming parameters and assembler that gave the best result for each phage DNA sample. Trimming was based on the output of FASTQC.

Sample	Trimming	Assembler
Escherichia coli	removed nucleotides from the right (5' end) according to quality score (min 20)<br>removed reads according to the mean quality (min 20)<br>removed reads shorter than 50 bp<br>remove reads with streaks of N longer than 10<br>removed 20 nucleotides on the left (3' end)<br>removed 10 nucleotides on the right (5' end)<br>removed duplicate reads	Genovo
Enterococcus	removed nucleotides from the right (5' end) according to quality score (min 20)<br>removed reads according to the mean quality (min 20)<br>removed reads shorter than 50 bp<br>remove reads with streaks of N longer than 10<br>removed 30 nucleotides on the left (3' end)<br>removed 10 nucleotides on the right (5' end)<br>removed duplicate reads	Genovo
Pseudomonas aeruginosa Pao1	removed nucleotides from the right (5' end) according to quality score (min 20)<br>removed reads according to the mean quality (min 20)<br>removed reads shorter than 50 bp<br>remove reads with streaks of N longer than 10<br>removed 20 nucleotides on the left (3' end)<br>removed 50 nucleotides on the right (5' end)<br>removed duplicate reads	Genovo
Pseudomonas aeruginosa 0407431-2	Untrimmed	Velvet
Proteus	Untrimmed	Velvet
Salmonella	removed nucleotides from the right (5' end) according to quality score (min 20)<br>removed reads according to the mean quality (min 20)<br>removed reads shorter than 36 bp<br>remove reads with streaks of N longer than 10	Genovo
Shigella flexneri	removed nucleotides from the right (5' end) according to quality score (min 20)<br>removed reads according to the mean quality (min 20)<br>removed reads shorter than 50 bp<br>remove reads with streaks of N longer than 10<br>removed 20 nucleotides on the left (3' end)<br>removed 10 nucleotides on the right (5' end)<br>removed duplicate reads	Genovo
Shigella sonnei	removed nucleotides from the right (5' end) according to quality score (min 20)<br>removed reads according to the mean quality (min 20)<br>removed reads shorter than 50 bp<br>remove reads with streaks of N longer than 10<br>removed 20 nucleotides on the left (3' end)<br>removed 10 nucleotides on the right (5' end)<br>removed duplicate reads	Genovo

Table S2. Overview of the bacterial strains used for small scale susceptibility testing. Observe that all *Salmonella* are of the species *Salmonella enterica* subsp. *enterica* but they are identified as different serovars. All strains are part of an in-house collection.

Genus	Species/Serovar	Strain	Susceptibility
Salmonella	serovar Enteritidis	ATCC 13076 *,+	Yes
	serovar Typhimurium	ATCC 14028 *,+	Yes
	serovar Saint Paul	DVL31 +	Yes
	serovar Newport	EQAS1 98-24475-1 +	Yes
	serovar Infantis	EQAS1 98-74091-5 +	Yes
	serovar Derby	EQAS2 99-65209-5 +	Yes
	serovar Typhimurium	DT36 +	Yes
	serovar Enteritidis	PT1 +	Yes
	serovar Heidelberg	75-12893-1 +	Yes
	serovar Dublin	1111H11036 +	Yes
Staphylococcus	*aureus*	ATCC 29213 *,+	No
	aureus	ATCC 25923 *,+	Yes
	epidermidis	CCM2354	No
	pseudointermedius	Bjorn 55-4	No
	hyicus	NCTC 10350	No
	felis	Sneleopard	Yes
	lugdunensis	E2-1928945	No
	aureus	76670 CC8 related +	Yes
	aureus	MSSA +	Yes
	aureus	MSSA A7 +	Yes
Shigella	*flexneri*	1s +	Yes
	sonnei	2s +	Yes
	boydii	Not given +	Yes
	flexneri	Not given +	Yes
	Not given	HN-Sh, 2006-001, 2007-5-3 +	Yes
Pseudomonas	*aeruginosa*	DMS 1128/ATCC9027 *,~	No
	aeruginosa	Skejby_2 ~	No
	aeruginosa	07 52277-1 ~	Yes
	aeruginosa	PAOI seq ~	Yes
	aeruginosa	0173267-5 ~	Yes
	aeruginosa	0407431-2 ~	Yes
	aeruginosa	0107338-1 ~	Yes
Escherichia	*coli*	ATCC 25922 *	Yes
	coli	C 64-12 +	No
	coli	C 60-12 +	No
	coli	C 23-12 +	No
	coli	oedemsyge-45	No
	coli	BW25II3	Yes
Proteus	*hauseri*	DSM 30118/ATCC 13315 *,~	Yes
	vulgaris	DMS 2140/ATCC 8427 *,~	No
	vulgaris	CCUG 36761, ATCC 13315 *,~	Yes
	mirabilis	76499961 ~	Yes
	mirabilis	E2 1928244 ~	No
Enterococcus	*faecalis*	2011-70-7-6 to 2011-70-250-4 ~	No
	faecium	2011-70-7-8 to 2011-70-252-10 ~	Yes
	faecalis	2008-37857 ~	No
	faecalis	12 E ~	No
	faecalis	ATCC 29212 *,~	Yes

Notes: Reference strains are marked with an asterisk (*). Pathogenic strains are marked with a plus (+), opportunistic pathogens with a tilde (~).

Table S3. Overview of phage clusters identified in the sequencing data of the host-amplified samples. Note that many clusters are much smaller in size compared to the corresponding clusters in the full cocktail. Those clusters have likely not been amplified by that particular host. Some clusters, however, e.g., EntF2 and Pao1_new show, a great increase in size. This can be explained by the fact that those are infecting clusters (compare Table 4 in the text), which are in higher abundance in the host-amplified samples compared to their original numbers in the cocktail. Therefore, greater parts of those clusters could be recovered from the amplified samples.

Phage Cluster in Sample	Cluster Size in bp	Corresponding Cluster in Intesti	Size Ratio to Corresponding Cluster
Amplified on *Escherichia coli*			
Eco1	9737	D1	0.07
Eco2	3163	D2	0.04
Eco3	19,979	D3	0.23
Eco4	1043	D4	0.02
Eco5	7023	D5	0.05
Eco6	133,873	D6	1.64
Eco7	17,744	D7	0.30
Eco9	5487	D9	0.14
Eco10	39,747	D10	0.27
Eco11	4131	D11	0.07
Eco12	12,195	D12	0.20
Eco13	9105	D13	0.05
Eco14	185,358	D14	1.39
Eco15	7278	D15	0.17
Eco16	18,144	D16	0.39
Eco17	78,630	D17	1.91
Eco18	8603	D18	0.21
EcoP	41,317	Proteus phage	0.40
Amplified on *Enterococcus faecalis*			
Ent7	58,552	D7	1.01
Ent11	6268	D11	0.10
Ent13	5282	D13	0.03
Ent18	41,874	D18	1.02
EntF2	88,702	F2	7.73
Amplified on *Pseudomonas aeruginosa* PAO1_seq			
Pao1_6	9257	D6	0.11
Pao1_10	1477	D10	0.01
Pao1_12	538	D12	0.01
Pao1_F1	22,920	F1	1.65
Pao1_P	3075	Proteus phage	0.03
Pao1_new	45,478	-	19.01
Amplified on *Pseudomonas aeruginosa* 0407431-2			
PA0407_3	87,742	D3	1.00
Amplified on *Salmonella typhimurium*			
Sal3	515	D3	0.01
Sal6	19,359	D6	0.24
Sal7	574	D7	0.01
Sal13	1047	D13	0.01
Sal14	717	D14	0.01
Sal18	46,366	D18	1.13
SalF2	94,543	F2	8.24
SalP	670	Proteus phage	0.01

Table S3.*Cont.*

Phage Cluster in Sample	Cluster Size in bp	Corresponding Cluster in Intesti	Size Ratio to Corresponding Cluster
Amplified on *Shigella flexneri*			
ShiFl1	2402	D1	0.02
ShiFl2	4799	D2	0.06
ShiFl3	1357	D3	0.02
ShiFl6	21,797	D6	0.27
ShiFl7	3946	D7	0.07
ShiFl9	3362	D9	0.08
ShiFl10	1102	D10	0.01
ShiFl12	7707	D12	0.13
ShiFl13	1784	D13	0.01
ShiFl14	177,744	D14	1.34
ShiFl15	48,286	D15	1.10
ShiFl16	4765	D16	0.10
Amplified on *Shigella sonnei*			
ShiS2	6868	D2	0.09
ShiS6	11,588	D6	0.14
ShiS14	173,647	D14	1.31
ShiS15	49,031	D15	1.12
ShiS16	4075	D16	0.09
ShiSP	5715	Proteus phage	0.05
Amplified on *Proteus vulgaris*			
Prot17	59,325	D17	1.44
ProtP	102,963	Proteus phage	0.99

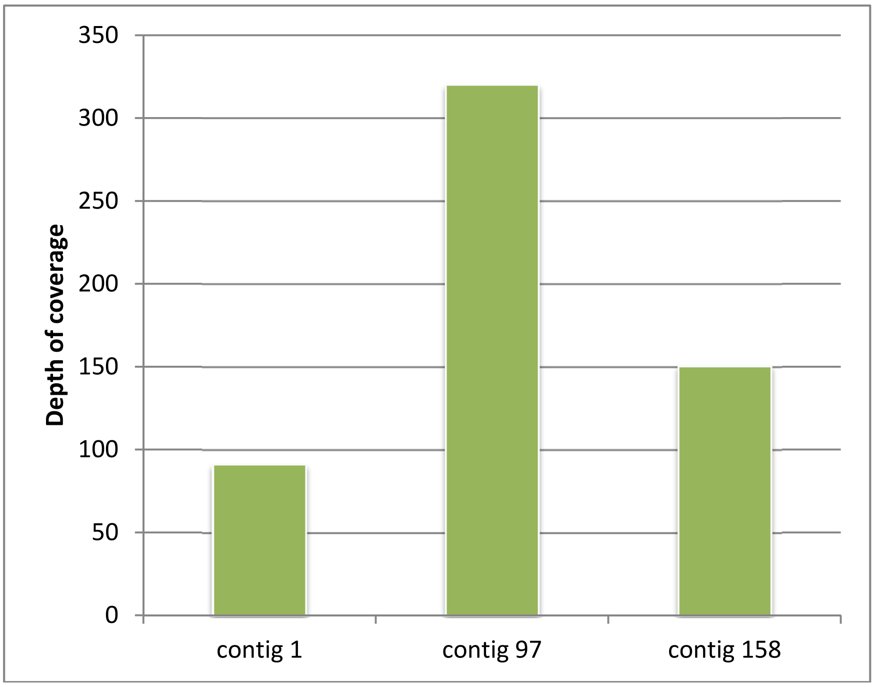

(**a**)

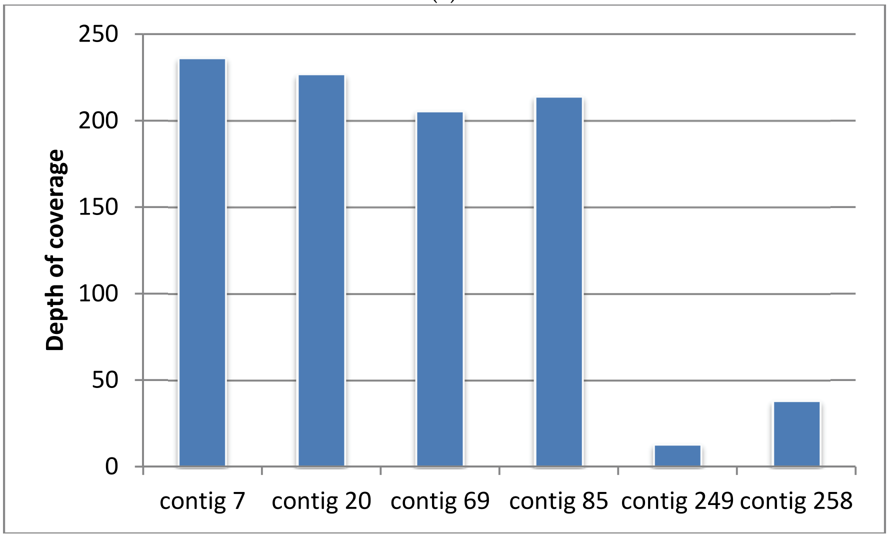

(**b**)

Figure S1. Two examples of clusters whose depth of coverage had a large standard deviation. The lower the contig ID, the longer the contig. (**a**) Depth of coverage of cluster D1. Contig 1, which is the longest, has a much lower depth of coverage than the short contigs, 97 and 158. Annotation results showed that many of the genes in contigs 97 and 158 show homology to genes annotated as "terminal repeat-encoded protein (Tre)"; (**b**) Depth of coverage of cluster D6. The two short contigs, 249 and 258, have much lower depth than the other contigs in that group. We theorize that they could represent divergent regions only present in a few of the phages in that cluster

© 2015 by the authors; licensee MDPI, Basel, Switzerland. This article is an open access article distributed under the terms and conditions of the Creative Commons by Attribution (CC-BY) license (http://creativecommons.org/licenses/by/4.0/).
